# Nonalcoholic fatty liver disease is associated with increased hemoconcentration, thrombocytopenia, and longer hospital stay in dengue-infected patients with plasma leakage

**DOI:** 10.1371/journal.pone.0205965

**Published:** 2018-10-17

**Authors:** Suhendro Suwarto, Riyanti Astrid Diahtantri, Mohammad Jauharsyah Hidayat, Bing Widjaya

**Affiliations:** 1 Tropical and Infectious Diseases Consultant, Pondok Indah Hospital, Jakarta, Indonesia; 2 Division of Tropical and Infectious Diseases, Department of Internal Medicine, Faculty of Medicine Universitas Indonesia, Cipto Mangunkusumo National Hospital (RSCM), Jakarta, Indonesia; 3 Faculty of Medicine, Universitas Indonesia, Jakarta, Indonesia; 4 Department of Radiology, Pondok Indah Hospital, Jakarta, Indonesia; 5 Department of Clinical Pathology, Pondok Indah Hospital, Jakarta, Indonesia; Monash University Malaysia, MALAYSIA

## Abstract

A prominent histopathological feature of fatal dengue cases is hepatic steatosis. However, the association between hepatic steatosis and dengue severity is unknown. We conducted a study to determine the associations of nonalcoholic fatty liver disease (NAFLD) with laboratory markers of dengue severity and length of hospital stay (LOS). A retrospective study was conducted at a private hospital in Jakarta, Indonesia, from December 2011 to December 2016. Bivariate analysis was performed to analyze the associations of laboratory markers of dengue severity and LOS with the presence or absence of NAFLD in no-plasma-leakage (no leakage) and plasma-leakage (leakage) groups. There were 267 dengue-infected patients included in this study. Of these patients, 115 (43.1%) were classified as belonging to the no leakage group, and 152 (56.9%) were classified as belonging to the leakage group. Of the 115 patients belonging to the no leakage group, 53 (46.1%) did not have NAFLD, and 62 (53.9%) had NAFLD. Of the 152 patients belonging to the leakage group, 85 (55.9%) did not have NAFLD, and 67 (44.1%) had NAFLD. Leakage group patients with NAFLD experienced significantly higher hemoconcentration severity (p = 0.04), lower platelet count (p = 0.004) and higher LOS (p = 0.042) than did leakage group patients without NAFLD. The presence of NAFLD in dengue-infected patients with plasma leakage was associated with more severe hemoconcentration, thrombocytopenia and prolonged hospital stay.

## Introduction

Dengue virus (DENV) belongs to the family Flaviviridae and the genus Flavivirus. There are four known DENV serotypes (DEN1−4) [1]. The clinical manifestations of dengue-infected patients are classified into dengue fever (DF), dengue with plasma leakage or dengue hemorrhagic fever (DHF) and dengue shock syndrome (DSS) [2]. Previous studies have reported that the abdominal ultrasound examination is highly sensitive and specific for determining plasma leakage and is superior to hemoconcentration or hypoalbuminemia testing [3–5]. In addition, laboratory parameters including aspartate aminotransferase (AST) level, alanine aminotransferase (ALT) level, platelet count, serum hematocrit, and serum albumin level are used as markers of dengue severity [3,6]. The associations between dengue severity and several different risk factors have been investigated previously. One risk factor that may be associated with dengue infection severity is obesity [7,8].

Obesity is considered a public health problem but was previously thought to be uncommon in Asia; however, the prevalence of obesity has increased throughout countries in Asia and the Pacific [9]. Evidence indicates that obesity is associated with nonalcoholic fatty liver disease (NAFLD) [10]. NAFLD is defined by hepatic steatosis without secondary causes of lipid accumulation in the liver, such as excessive alcohol consumption or steatogenic medications [11]. NAFLD patients are usually asymptomatic. The most common laboratory abnormality is elevated ALT levels exceeding AST levels. In clinical settings, NAFLD is diagnosed by abdominal ultrasonography [12]. A study in Indonesia, which has a high incidence of dengue infection [13], revealed that the prevalence of NAFLD in an urban population was approximately 30%, with obesity being the highest risk factor [14].

Several studies investigating severe dengue infection fatalities reported that the liver was the most commonly affected organ [15,16]. Postmortem histopathology indicated moderate to severe congestion of hepatic sinusoids with various degrees of cell death in the liver accompanied by fatty infiltration or steatosis in the hepatic lobules in almost all cases [17]. Autopsies revealed a significant difference in the presence of steatosis between dengue-infected patients and a non-dengue-infected group [15]. In vitro studies using DENV-infected cell models found that DENV regulates cellular lipid metabolism to enhance viral replication [18]. In addition, viral replication is associated with elevated liver transaminase levels, thrombocytopenia and dengue severity [19–21]. However, no study has investigated the associations between NAFLD and laboratory markers of dengue severity in infected patients. Knowledge of these associations is essential for evaluating potential risk factors for disease severity and their impacts in clinical practice. Thus, we investigated the associations of NAFLD with liver transaminase level, platelet count, degree of hemoconcentration, albumin level and length of hospital stay (LOS) in dengue-infected patients with and without plasma leakage.

## Materials and methods

A retrospective study was conducted at Pondok Indah Hospital in Jakarta, Indonesia, from December 2011 to December 2016. The following patient inclusion criteria were applied in this study: acute fever ≥3 days, age ≥14 years, tested positive for dengue nonstructural protein 1 (NS1) antigen (SD BIOLINE Dengue Duo, Standard Diagnostics, Korea) and underwent abdominal ultrasonography (USG). Clinical characteristics, body mass index (BMI), history of diabetes mellitus, laboratory parameters and USG were recorded. Daily complete blood counts were performed to determine lowest platelet count and degree of hemoconcentration. The degree of hemoconcentration was calculated using a previously published formula [3]. AST and ALT levels were recorded twice: once during the febrile phase and once during the critical phase. Measurements of albumin level and abdominal USG were carried out during the critical phase. The decision to perform abdominal USG was at physician discretion. The febrile phase was defined as day 1 to day 5 of fever, and the critical phase was defined as 1–2 days after defervescence [22]. Clinical manifestations of severe dengue and LOS were recorded. Severe dengue was defined based on WHO criteria [23]. LOS was calculated by subtracting the patient's admission date from his/her discharge date.

Abdominal ultrasound was performed by a certified radiologist using an ultrasound system (Acuson S 2000, Siemens, Germany) with convex transducers (6C1 HD, frequency bandwidth 1.5–6.0 MHz). The radiologist report included routinely examined organs, i.e., liver, bile ducts, gallbladder, pancreas, spleen, kidney, ascites/pleural effusion and gynecologic ultrasound. Fatty liver was diagnosed by liver echogenicity of the ultrasonogram that exceeded that of the renal cortex [24]. USG results were recorded as the presence or absence of fatty liver and the presence or absence of pleural effusion and/or ascites. Patients without pleural effusion and/or ascites were classified as having no plasma leakage (no leakage), and those with pleural effusion and/or ascites were classified as having plasma leakage (leakage). The exclusion criteria were incomplete data, medications that can cause hepatotoxicity, consumption of herbal products, pre-existing chronic liver disease, excessive alcohol consumption (2 standard drinks per day for men and one standard drink per day for women) [25], and pregnancy. To evaluate the associations between NAFLD and dengue severity markers, patients were categorized into the following four groups: group 1 comprised patients in the no leakage group who did not have NAFLD (no leakage-NAFLD), group 2 comprised patients in the no leakage group who had NAFLD (no leakage+NAFLD), group 3 comprised patients in the leakage group who did not have NAFLD (leakage-NAFLD), and group 4 comprised patients in the leakage group who had NAFLD (leakage+NAFLD).

We calculated sample size based on the difference between two population mean formulas that require estimates of population variance. Population variance was calculated from the standard deviation. The standard deviations of dengue severity markers (AST level, ALT level, hemoconcentration, lowest platelet count, and albumin level) were calculated from 10 patients in four preliminary study groups [26]. Based on our preliminary study, the albumin population variance was 0.251, and the hypothesized difference was 0.25. At α = 0.05 and β = 0.20, the minimum total sample size for the four groups was 256 patients. This total sample size was the highest sample size required for all analyzed dengue severity markers.

We used the Mann-Whitney test for nonparametric data and the chi-square test or Fisher’s exact test for categorical variables. Statistical analyses were performed using STATA Statistics Data Analysis version 14.2 (Stata Corp., College Station, TX, USA).

### Ethics statement

The Faculty of Medicine Universitas Indonesia Ethics Committee and the management at Pondok Indah Hospital Jakarta approved this study and waived the requirement for informed consent. All the patients' data were fully anonymized prior to analysis.

## Results

### Clinical characteristics of dengue-infected patients

**Fig 1:**
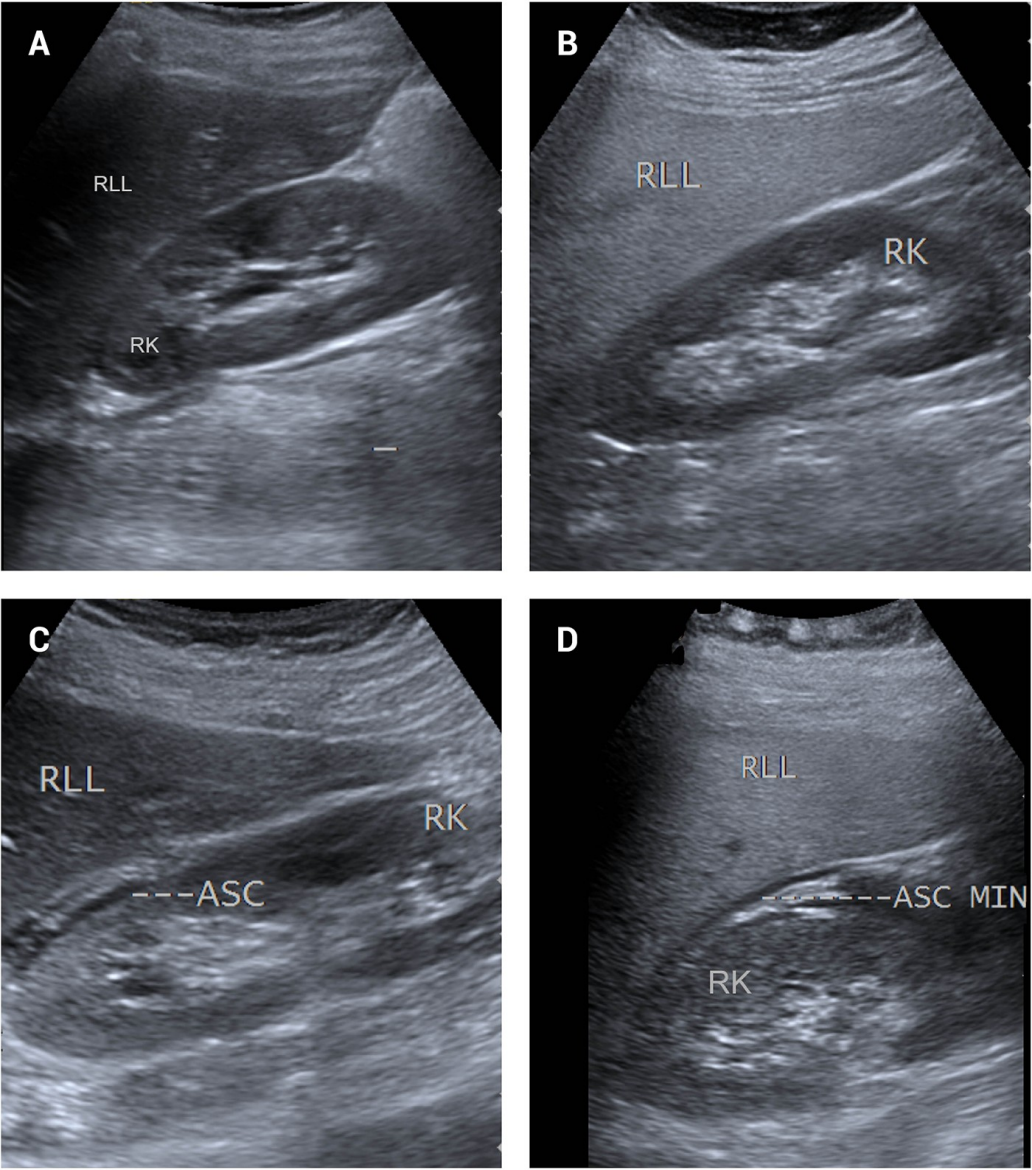
Ultrasonography findings of the four groups studied. (A) No leakage-NAFLD group: Normal liver echogenicity without pleural effusion/ascites. (B) No leakage+NAFLD group: Increased liver echogenicity relative to the renal parenchyma without pleural effusion/ascites. (C) Leakage-NAFLD group: Normal liver echogenicity with ascites. (D) Leakage+NAFLD group: Increased liver echogenicity relative to the renal parenchyma with ascites. Abbreviations: A: ascites; RK: right kidney; RLL: right liver lobe; MIN: minimal.

This study included 267 dengue-infected patients with a median age of 34 years (interquartile range = 24−45), and 155 patients (58.1%) were male. Of the 267 patients, 115 (43.1%) were classified as having no leakage, and 152 (56.9%) were classified as having leakage. Eight patients (2.99%) were classified as having severe dengue. No deaths occurred during this study. Table 1 presents the patients’ clinical characteristics. Fig 1 presents the USG findings of four groups studied.

**Table 1:** Clinical characteristics of the no leakage and leakage groups.

	No leakage group (n = 115)	Leakage group (n = 152)
Sex, no. female/male	42/73	70/82
Age, year, median (IQR)	35 (24–47)	32.5 (25–43.75)
BMI, kg/m^2^, median (IQR)	24.22 (22.83–27.88)	24.60 (23.04–26.62)
Blood pressure, critical phase		
SBP, mmHg, median (IQR)	110 (110–120)	110 (110–120)
DBP, mmHg, median (IQR)	80 (70–80)	73 (70–80)
AST level, febrile phase, U/L, median (IQR)	41 (27–65)	49 (33–89.50)^a^^,^*
ALT level, febrile phase, U/L, median (IQR)	54 (35–84)	56 (37–88.75)
AST level, critical phase, U/L, median (IQR)	81 (43–133)	152.50 (94.25–279.5) ^a^^,^*
ALT level, critical phase, U/L, median (IQR)	83 (51–133)	119 (69–218.25) ^a^^,^*
Hemoconcentration, %, median (IQR)	10.62 (7.71–13.85)	16.92 (11.25–23.09) ^a^^,^*
Lowest platelet count, × 1,000/μL, median (IQR)	69 (34–101)	19 (12–33.75) ^a^^,^*
Albumin level, g/dL, median (IQR)	3.5 (3.2–3.7)	3.1 (2.9–3.3) ^a^^,^*
Absence of NAFLD, n (%)	53 (38.4)	85 (61.6)
Presence of NAFLD, n (%)	62 (48.1)	67 (51.9)
Severe dengue, n (%)	0	8 (5.26)^b^^,^*
AST or ALT ≥1000 U/L, n (%)	0	3 (1.97)
Shock, n (%)	0	2 (1.31)
Respiratory distress, n (%)	0	1 (0.66)
Acute kidney injury, n (%)	0	1 (0.66)
Multiple organ failure, n (%)	0	1 (0.66)
LOS, days, median (IQR)	5 (4–6)	6 (5–7) ^a^^,^*

Abbreviations: BMI, body mass index; SBP, systolic blood pressure; DBP, diastolic blood pressure; AST, aspartate aminotransferase; ALT, alanine aminotransferase; IQR, interquartile range; NAFLD, nonalcoholic fatty liver disease; LOS, length of hospital stay

^a^ The Mann-Whitney test was used to assess differences between the no leakage and leakage groups.

^b^ Fisher’s exact test was used to evaluate associations between the no leakage and leakage groups.

* Significantly different from the no leakage group (p<0.05).

### Differences in clinical characteristics between patients belonging to the no leakage and leakage groups

There were significant differences in AST level during the febrile phase (p = 0.006) and critical phase (p = <0.001), ALT levels during the critical phase (p<0.001), degree of hemoconcentration (p<0.001), lowest platelet count (p<0.001), albumin level during the critical phase (p<0.001), severe dengue (p = 0.011) and LOS (p<0.001) between the no leakage and leakage groups (Table 1).

### BMI, laboratory parameters and LOS in patients with and without NAFLD

In the absence or presence of plasma leakage, BMI was higher in patients with NAFLD than in those without (p<0.001 for each comparison) (Table 2 and Table 3). In the absence of plasma leakage, the only laboratory marker of disease severity that was significantly different between patients with and without NAFLD was ALT level during the febrile phase (p = 0.042) (Table 2). In the presence of plasma leakage, when comparing patients with or without NAFLD, there were significant differences observed in ALT level during the febrile phase (p = 0.005), degree of hemoconcentration in the critical phase (p = 0.04), lowest platelet count in the critical phase (p = 0.004), and LOS (p = 0.042) (Table 3).

**Table 2:** BMI, laboratory parameters and LOS in the no leakage group: NAFLD-absent and NAFLD-present groups.

	No leakage-NAFLD group (n = 53)	No leakage+NAFLD group (n = 62)
BMI, kg/m^2^, median (IQR)	23.30 (21.33–24.34)	25.71 (23.81–28.46)*
AST level, febrile phase, U/L, median (IQR)	40 (25–61.50)	43.5 (28.75–68.5)
ALT level, febrile phase, U/L, median (IQR)	49 (30–71.50)	59 (43.50–86)*
AST level, critical phase, U/L, median (IQR)	79 (41–125)	82 (47.25–135)
ALT level, critical phase, U/L, median (IQR)	81 (44.50–139)	86.5 (57.25–125.5)
Hemoconcentration, %, median (IQR)	10.32 (7.55–13.71)	11.02 (8.21–13.92)
Lowest platelet count, × 1,000/μL, median (IQR)	73 (37.5–100)	66.5 (34–109.75)
Albumin level, g/dL, median (IQR)	3.5 (3.3–3.65)	3.4 (3.2–3.7)
LOS, days, median (IQR)	5 (4–6)	5 (4–6.25)

Abbreviations: NAFLD, nonalcoholic fatty liver disease; BMI, body mass index; AST, aspartate aminotransferase; ALT, alanine aminotransferase; IQR, interquartile range; LOS, length of hospital stay

The Mann-Whitney test was performed to assess differences in BMI, laboratory parameters and LOS between groups with and without NAFLD.

* Significantly different from patients in the no leakage-NAFLD group (p<0.05).

**Table 3:** BMI, laboratory parameters and LOS in the leakage group: NAFLD-absent and NAFLD-present groups.

	Leakage-NAFLD group (n = 85)	Leakage+NAFLD group (n = 67)
BMI, kg/m^2^, median (IQR)	23.73 (22.13–25.24)	25.95 (23.87–28.08)*
AST level, febrile phase, U/L, median (IQR)	48 (31.50–98)	50 (34–85)
ALT level, febrile phase, U/L, median (IQR)	45 (33.50–82)	65 (44–97)*
AST level, critical phase, U/L, median (IQR)	147 (81.50–288.50)	154(104–275)
ALT level, critical phase, U/L, median (IQR)	114 (57.50–242)	132 (81–208)
Hemoconcentration, %, median (IQR)	15.62 (10.25–21.48)	18.77 (11.87–24.80)*
Lowest platelet count, × 1,000/μL, median (IQR)	23 (13.5–40)	16 (10–25)*
Albumin level, g/dL, median (IQR)	3.1 (2.9–3.35)	3.1 (2.9–3.3)
LOS, days, median (minimum-maximum; IQR)	6 (2–14; 5–7)	6 (3–43; 5–7)*

Abbreviations: NAFLD, nonalcoholic fatty liver disease; BMI, body mass index; AST, aspartate aminotransferase; ALT, alanine aminotransferase; IQR, interquartile range; LOS, length of hospital stay

The Mann-Whitney test was performed to assess differences in BMI, laboratory parameters and LOS between groups with and without NAFLD.

* Significantly different from patients in the leakage-NAFLD group (p<0.05).

## Discussion

This is the first Indonesian study to assess the associations between NAFLD and markers of disease severity, including serum AST, ALT and albumin levels, platelet counts, and hematocrit findings. Plasma leakage is a severe form of dengue infection that occurs when intravascular fluid moves toward the extravascular compartment [27]. DENV infection with plasma leakage has been reported to be associated with severe dengue and prolonged hospital stays [6,28]. The factors that contribute to plasma leakage have not been fully explored; however, it has been suggested that complex interactions among virus factors and host factors play roles in the development of plasma leakage [29].

In this study, we found that there were significant differences in various laboratory markers of disease severity, excluding ALT level during the febrile phase, between the no leakage and leakage groups. There are several possible mechanisms by which hepatic transaminase levels can be increased during dengue infection; these include direct effects of DENV, host immune responses to the virus in hepatic cells, and hypoxia due to microvascular leakage or DSS [30,31]. Serum AST levels immediately increased following dengue infection, and this change was followed by an increase in ALT levels within 24 to 48 hours. ALT levels reach their peak during the critical phase [30,32]. These findings may explain why no difference in ALT level was observed between the two groups during the febrile phase, whereas a significant difference was observed during the critical phase.

The results of this study show that NAFLD is associated with higher BMI. High BMI is a risk factor for obesity-associated NAFLD [33]. Our findings regarding ALT levels in the no leakage and leakage groups suggest that the increase in ALT level that occurs during dengue infection is delayed. However, during the febrile phase, in the no leakage group, ALT levels were significantly higher in patients with NAFLD than in those without. This finding can be explained as follows. First, the most common laboratory abnormality in NAFLD is elevated hepatic transaminase as an inflammatory response to triglyceride accumulation in the liver parenchyma, which results from increased lipolysis of adipose tissue due to insulin resistance or excess dietary fat [34]. Second, the plasma half-life of ALT in the blood is longer than the plasma half-life of AST [32]. Third, in NAFLD, serum ALT is more commonly elevated than is AST, and it is often used as a surrogate marker of NAFLD [35,36]. In contrast, while ALT levels were higher in patients with NAFLD during the febrile phase, there were no significant differences in other laboratory markers of disease severity between the no leakage-NAFLD and no leakage+NAFLD groups.

DENV infection without plasma leakage is classified as mild dengue infection or DF [3]. The clinical findings observed in DF patients include mild thrombocytopenia (nadir platelet counts greater than 50,000/μL), mild hematocrit increases (increases in hemoconcentration of less than 15%), AST or ALT level less than 2.5 times the upper limit of normal, generally normal serum albumin level (≥3.4 g/dL) and LOS ≤5 days [3,28,37]. These clinical findings are similar to our results in both the no leakage-NAFLD and no leakage+NAFLD groups. These present and previous findings may explain why laboratory markers of dengue severity (excluding ALT level during the febrile phase) and LOS were not significantly different between the no leakage-NAFLD and no leakage+NAFLD groups in this study.

The laboratory results in the no leakage group suggest that the significantly different ALT levels observed during the febrile phase are attributable to NAFLD. Furthermore, the absence of any differences in other laboratory markers of disease severity between the no leakage-NAFLD and no leakage+NAFLD groups may be because no leakage is a mild form of dengue infection.

As previously described, NAFLD played a role in elevating ALT levels in the no leakage group during the febrile phase. ALT levels were also significantly higher in the leakage+NAFLD group than in the leakage-NAFLD group during the febrile phase. In contrast, there were no significant differences in serum ALT level during the critical phase or in serum AST level during the febrile or critical phase between the leakage-NAFLD and leakage+NAFLD groups. A previous study reported that AST and ALT levels were significantly higher in DHF patients with multiple organ failure than in DHF patients without multiple organ failure [38]. In contrast, in the present study, only 1 patient in the leakage group had multiple organ failure.

Compared to leakage group patients without NAFLD, patients belonging to the leakage group with NAFLD were significantly associated with severe hemoconcentration. There are several possible underlying mechanisms that could explain this finding. First, previous in vitro studies reported that lipid droplets in cells are important for DENV replication [39]. Moreover, postmortem studies of dengue fatalities revealed that lipid vesicles in liver cells appear to be used for viral replication [15]. The binding of DENV to endothelial cells and the immunopathogenic response caused by higher viral replication rates lead to increased endothelial permeability, which contributes to a higher hemoconcentration [40,41]. Second, elevated serum levels of free fatty acids (FFAs) and insulin resistance were associated with the development of hepatic steatosis [42]. The persistent accumulation of FFAs in the liver results in the release of pro-inflammatory cytokines that contribute to low-grade chronic inflammation [43]. In vitro studies performed using intestinal epithelial cell models have reported that pro-inflammatory cytokines increase epithelial permeability via paracellular pathways by disrupting tight junctions [44]. It has been suggested that high FFA levels that disrupt intercellular junctions and enhance paracellular permeability contribute to endothelial dysfunction [45]. Furthermore, in DENV infection, the increased degradation of tight junctions was found to be associated with severe plasma leakage [27]. When DENV infection occurs in NAFLD patients who already have endothelial dysfunction, it may increase the severity of plasma leakage and contribute to more severe hemoconcentration [46].

Additionally, patients in the leakage group with NAFLD had significantly more severe thrombocytopenia than did those without NAFLD. A previous study reported that the thrombocytopenia observed in DENV patients may be due to the increased adherence of platelets to sites of vascular endothelial injury [47]. The increased endothelial injury in DENV infection results in plasma leakage [48]. As previously discussed, when DENV infection occurs in NAFLD patients, it may increase the severity of plasma leakage. In cases with more severe plasma leakage, platelet adherence may lower nadir platelet counts [46]. Finally, DENV infection with more severe hemoconcentration and thrombocytopenia results in longer hospital stays [28].

We observed no significant difference in serum albumin levels between NAFLD-absent and -present patients in the leakage group. According to previous studies, vascular leakage caused by DHF decreases serum albumin levels to 3–3.5 g/dl [27,49]. Furthermore, during severe dengue infection, albumin leakage results in decreased serum albumin levels of ≤3 g/dl [27,50]. In contrast to these previous studies, our study found that in the leakage group in our study, only 5.26% of patients had severe dengue. This may explain the lack of a significant difference in albumin levels between the NAFLD-absent and NAFLD-present patients.

The results of our study emphasize that obesity-associated NAFLD increases the severity of dengue infection in patients with plasma leakage. This finding is consistent with a recent study that reported that obesity is significantly associated with increased hematocrit, decreased platelet count and longer hospitalization in dengue-infected patients [46].

This study has several limitations. First, there was a lack of liver biopsy in this study, which is the gold standard for diagnosing NAFLD. Liver biopsies are also used to detect the progressive form of NAFLD, nonalcoholic steatohepatitis (NASH) or steatosis with inflammation and hepatocyte injury. However, this procedure is highly invasive and thus is not recommended in dengue studies due to thrombocytopenia. Second, we did not assess comorbidities, such as cardiovascular disease, chronic kidney disease and chronic liver disease, and this limits the generalizability of the present results to other populations.

In conclusion, in dengue-infected patients with plasma leakage, the presence of NAFLD was associated with more severe hemoconcentration, lower platelet counts and increased LOS. Further studies are required to investigate the role of NAFLD in the most severe dengue cases, including patients with fatal dengue hemorrhagic fever.

## Supporting information

S1 Table Comparison between the no leakage and leakage groups: Clinical characteristics and laboratory parameters. Abbreviations: BMI, body mass index; SBP, systolic blood pressure; DBP, diastolic blood pressure; ALT, alanine aminotransferase; IQR, interquartile range; NAFLD, non-alcoholic fatty liver disease. ^a^ The Mann-Whitney test was used to assess differences between the no leakage and leakage groups. ^b^ Chi-square tests was used to evaluate associations between the no leakage and leakage groups. ^c^ Fisher’s exact tests was used to evaluate associations between the no leakage and leakage groups.
(DOCX)

S2 Table Differences between the absence and presence of NAFLD in the no leakage groups. Abbreviations: NAFLD, non-alcoholic fatty liver disease; AST, aspartate aminotransferase; ALT, alanine aminotransferase; IQR, interquartile range; LOS, length of hospital stay. Statistical analysis was assessed by Mann-Whitney test.
(DOCX)

S3 Table Differences between the absence and presence of NAFLD in the leakage groups. Abbreviations: NAFLD, non-alcoholic fatty liver disease; AST, aspartate aminotransferase; ALT, alanine aminotransferase; IQR, interquartile range. Statistical analysis was assessed by Mann-Whitney test.
(DOCX)
