# Supplementary material for: Nonalcoholic fatty liver disease is associated with increased hemoconcentration, thrombocytopenia, and longer hospital stay in dengue-infected patients with plasma leakage
Source: PLoS One. 2018 Oct 17;13(10):e0205965. doi: 10.1371/journal.pone.0205965 (PMC6192651; doi:10.1371/journal.pone.0205965)
Supplement: S1 Table — Abbreviations: BMI, body mass index; SBP, systolic blood pressure; DBP, diastolic blood pressure; ALT, alanine aminotransferase; IQR, interquartile range; NAFLD, non-alcoholic fatty liver disease. a The Mann-Whitney test was used to assess differences between the no leakage and leakage groups. b Chi-square tests was used to evaluate associations between the no leakage and leakage groups. c Fisher’s exact tests was used to evaluate associations between the no leakage and leakage groups. (DOCX) [file pone.0205965.s001.docx]

**S1 Table. Comparison between the No Leakage and Leakage Groups: Clinical characteristics and laboratory parameters**

|  | No leakage group (n=115) | Leakage group (n=152) | p |
| --- | --- | --- | --- |
| BMI | 24.22 (22.83-27.88) | 24.60 (23.04- 26.62) | 0.96^a^ |
| Blood pressure |  | | |
| SBP, mmHg, median (IQR) | 110 (110-120) | 110 (110-120) | 0.88^a^ |
| DBP, mmHg, median (IQR) | 80 (70-80) | 73 (70-80) | 0.87 ^a^ |
| Diabetes mellitus, n (%) | 4 (3.47) | 3 (1.97) | 0.46^c^ |
| ALT levels, febrile phase, U/L, median (IQR) | 54 (35-84) | 56 (37-88.75) | 0.63^a^ |
| Absence of NAFLD, n (%) | 53 (38.4) | 85 (61.6) | 0.14^b^ |
| Presence of NAFLD, n (%) | 62 (48.1) | 67 (51.9) |  |

Abbreviations: BMI, body mass index; SBP, systolic blood pressure; DBP, diastolic blood pressure; ALT, alanine aminotransferase; IQR, interquartile range; NAFLD, non-alcoholic fatty liver disease. ^a^ The Mann-Whitney test was used to assess differences between the no leakage and leakage groups. ^b^ Chi-square tests was used to evaluate associations between the no leakage and leakage groups. ^c^ Fisher’s exact tests was used to evaluate associations between the no leakage and leakage groups.
