# Supplementary material for: Nonalcoholic fatty liver disease is associated with increased hemoconcentration, thrombocytopenia, and longer hospital stay in dengue-infected patients with plasma leakage
Source: PLoS One. 2018 Oct 17;13(10):e0205965. doi: 10.1371/journal.pone.0205965 (PMC6192651; doi:10.1371/journal.pone.0205965)
Supplement: S3 Table — Abbreviations: NAFLD, non-alcoholic fatty liver disease; AST, aspartate aminotransferase; ALT, alanine aminotransferase; IQR, interquartile range. Statistical analysis was assessed by Mann-Whitney test. (DOCX) [file pone.0205965.s003.docx]

**S3 Table. Differences between the absence and presence of NAFLD in the Leakage Groups.**

|  | p |
| --- | --- |
| AST levels, febrile phase, U/L, median (IQR) | 0.71 |
| AST levels, critical phase, U/L, median (IQR) | 0.84 |
| ALT levels, critical phase, U/L, median (IQR) | 0.26 |
| Albumin levels, g/dL, median (IQR) | 0.97 |

Abbreviations: NAFLD, non-alcoholic fatty liver disease; AST, aspartate aminotransferase; ALT, alanine aminotransferase; IQR, interquartile range. Statistical analysis was assessed by Mann-Whitney test.
